# Extended X-Ray Absorption Fine Structure of ZrW_2_O_8_: Theory vs. Experiment

**DOI:** 10.3389/fchem.2018.00356

**Published:** 2018-08-23

**Authors:** Fernando D. Vila, John W. Spencer, Joshua J. Kas, John J. Rehr, Frank Bridges

**Affiliations:** ^1^Department of Physics, University of Washington, Seattle, WA, United States; ^2^Department of Physics, University of California, Santa Cruz, Santa Cruz, CA, United States

**Keywords:** zirconium tungstate, EXAFS, DFT, FEFF9, Debye-Waller factors

## Abstract

Extended x-ray absorption fine structure (EXAFS) is well-suited for investigations of structure and disorder of complex materials. Recently, experimental measurements and analysis of EXAFS have been carried out to elucidate the mechanisms responsible for the negative thermal expansion (NTE) in zirconium tungstate (ZrW_2_O_8_). In contrast to previous work suggesting that transverse O-displacements are largely responsible, the EXAFS analysis suggested that correlated rotations and translations of octahedra and tetrahedra within the structure are a major source. In an effort to resolve this controversy, we have carried out *ab initio* calculations of the structure, lattice vibrations, and EXAFS of ZrW_2_O_8_ based on real-space multiple-scattering calculations using the FEFF9 code and auxiliary calculations of structure and Debye-Waller factors. We find that the theoretical simulations are consistent with observed EXAFS, and show that both of the above mechanisms contribute to the dynamical structure of ZrW_2_O_8_.

## 1. Introduction

**Figure 1:**
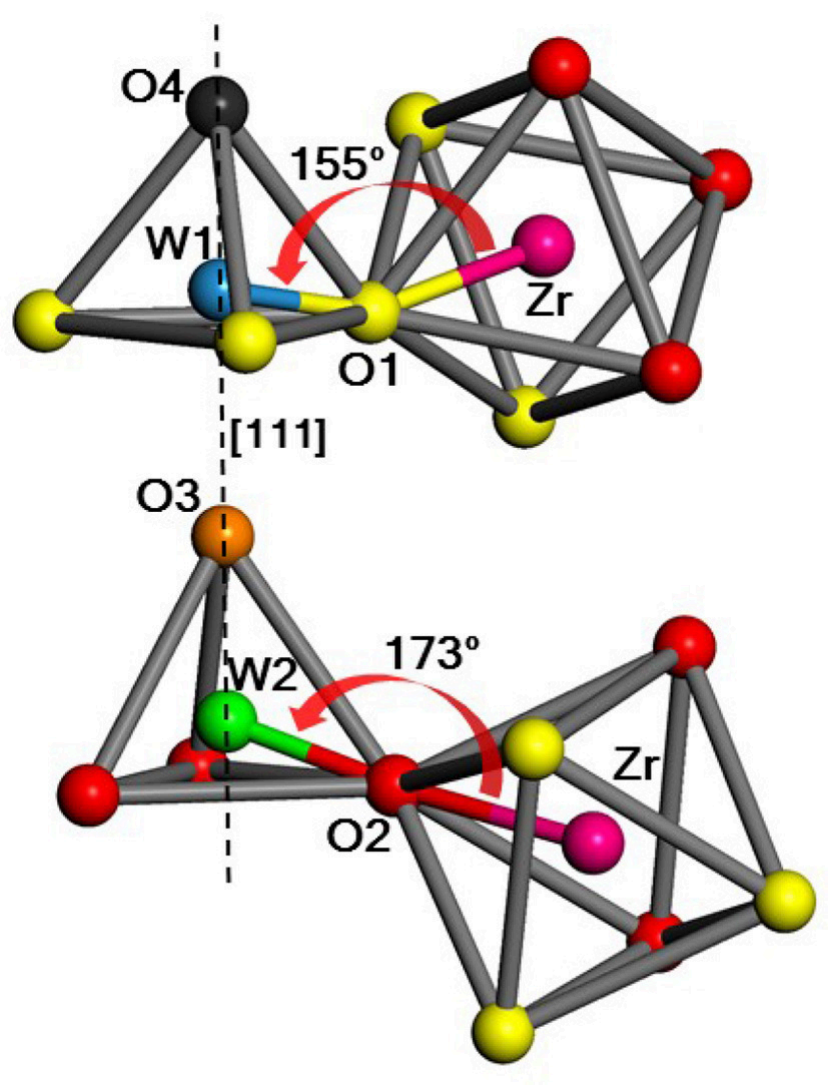
Portion of the unit cell showing the two possible WO_4_–ZrO_6_ linkages in ZrW2O8, with Zr (magenta), W1 (blue) and W2 (green), O1 (yellow), O2 (red), O3 (orange), O4 (black). W1, O1, and W2, O2 are the atoms that form Zr-O-W linkages with bridging angles of 155° and 173°, respectively, while the O3 and O4 atoms are only weakly linked (Table 1). [111] denotes the body diagonal direction of the unit cell.

Zirconium tungstate (ZrW_2_O_8_) is remarkable for its large negative thermal expansion (NTE). Unlike other such materials, ZrW_2_O_8_ exhibits NTE over a wide range of temperatures from about 10 to 1,050 K. However, the microscopic mechanism(s) underlying its NTE is controversial. In several studies this has been associated primarily with the transverse motion of the central O atom in the Zr-O-W linkages (Figure 1).

Recently, however, an alternative mechanism based on correlated translations of the W-centered oxygen tetrahedra and rotations of Zr-centered oxygen octahedra has been proposed (Cao et al., 2002, 2003; Bridges et al., 2014). This interpretation is based on experimental investigations of ZrW_2_O_8_ using extended x-ray absorption fine structure (EXAFS), a technique well suited for eludicating the structure and disorder in complex materials. X-ray total scattering results are consistent with this interpretation (Bridges et al., 2014). In particular these analyses suggest that the Zr-O2-W2 linkages are quite stiff, with the implication of limited transverse O motion. In an effort to resolve the controversy, we have carried out *ab initio* simulations of EXAFS of ZrW_2_O_8_ using the real-space multiple-scattering (RSMS) code FEFF9 (Rehr et al., 2009) and associated Debye-Waller factors (Vila et al., 2007). We find that these calculations are consistent with the observed EXAFS, and show that both of the above mechanisms contribute to the dynamical structure of ZrW_2_O_8_.

In the remainder of this work we first summarize the lattice structure of ZrW_2_O_8_, the multiple-scattering theory of EXAFS, and the theory of the multiple-scattering Debye-Waller factors. Subsequently we describe the results of our simulations, followed by a summary and conclusions.

## 2. Theoretical considerations

### 2.1. Structure of ZrW_2_O_8_

The first step in our approach is a detailed treatment of the ground state equilibrium structure of ZrW_2_O_8_. Our calculations assume a periodic equilibrium structure with a 44-atom unit cell enclosing four equivalent Zr, 8 W, and 32 O atoms. The metal atoms are centered in polyhedra with O vertices: octahedra for Zr, and tetrahedra for W. The O atoms shared between the Zr-octahedra and W-tetrahedra form Zr–O–W “linkages.” This detailed structure was refined with *ab initio* DFT calculations using the VASP plane-wave pseudopotential code (Kresse and Hafner, 1993, 1994; Kresse and Furthmüller, 1996a,b; Kresse and Joubert, 1999). These calculations show that the structure has a large degree of asymmetry; the octahedra are slightly irregular, and the unit cell contains Zr-O-W bond linkages with equally populated bond angles of about 155 and 173° respectively (see Figure 1 and Table 1).

**Table 1:** Experimental and theoretical structural parameters for the 44-atom ZrW_2_O_8_ cell used in this work.

		**Expt**.	**DFT**
Lattice parameter		9.155	9.246
Bonds	W1–O1	1.799	1.826
	W1–O4	1.712	1.728
	W2–O2	1.785	1.797
	W2–O3	1.736	1.751
	Zr–O1	2.051	2.062
	Zr–O2	2.092	2.119
	Zr–W1	3.751	3.795
	Zr–W2	3.867	3.909
Angles	Zr–O1–W1	153.9	155.0
	Zr–O2–W2	171.5	173.2

*All distances are in Å and angles in degrees; experimental values are taken from Auray et al. (1995), while the DFT calculations are done using VASP with a PBEsol exchange-correlation functional*.

### 2.2. Multiple-scattering EXAFS theory

Formally, the normalized EXAFS at a given photon energy *E* is defined as χ = (μ−μ_0_)/μ_0_, where μ is the x-ray absorption coefficient and μ_0_ the smooth atomic background. Theoretically χ can be represented by a sum over all possible single- (SS) and multiple-scattering (MS) paths *p* taken by the photoelectron following photo-absorption (Rehr et al., 2009). Assuming the thermal and structural disorder is sufficiently small, χ(*k*) is given by the full path expansion

(1a) $$\begin{array}{l} \chi \left(k\right)=\underset{p}{\sum }N_{p}\chi_{p}\left(k\right)e^{-2\sigma_{p}^{2}k^{2}}, \end{array}$$

(1b) $$\begin{array}{l} \chi_{p}\left(k\right)=S_{0}^{2}\frac{\left|f_{\text{eff}}^{p}\left(k\right)\right|}{kR_{p}^{2}}\sin\left(2kR_{p}+\Phi_{p}\right)e^{-2R/\lambda_{k}}. \end{array}$$

Here $k=\sqrt{2\left(E-E_{0}\right)}$ is the wave-number of the photoelectron defined relative to threshold energy *E*_0_, $f_{\text{eff}}^{p}\left(k\right)$ the effective scattering amplitude of path *p*, Φ_*p*_ the net scattering phase-shift, *N*_*p*_ the degeneracy of the path, *R*_*p*_ the effective- (or half-)path length, $S_{0}^{2}$ is the many-body amplitude factor, typically close to unity and path independent, λ_*k*_ is the mean free path, and $\sigma_{p}^{2}$ is the mean-square relative displacement (MSRD) of path *p*, which characterizes the path length fluctuations. Unless otherwise specified we use atomic units for the electron, *e* = ℏ = *m* = 1.

In this paper we are primarily interested in the EXAFS from the Zr K and W L_3_ edges, which are relevant to previous EXAFS studies of NTE in ZrW_2_O_8_. In particular our study focuses on the contributions to EXAFS from the SS and MS paths linking a given Zr atom to a nearby W atom or vice versa. These paths are denoted by the scattering atoms in the path. For example, the paths Zr-W-Zr, Zr-W-O-Zr, and Zr-O-W-O-Zr also have the highest *amplitude importance ratios*, which are defined relative to the amplitudes for the shortest Zr-O or W-O paths. As discussed in our companion study (Vila et al., 2018), the NTE in ZrW2O8 can be linked to anharmonic low frequency modes with negative Grüneisen parameters. This effect is strongest for modes with transverse O-displacements, which can also involve correlated rotations and translations. Thus we also discuss the effects of such transverse motion. Details of the dominant paths including averages of effective path lengths weighted by path degeneracy are listed in Table 2, where (W1, O1) and (W2, O2) denote the W and O atoms in the Zr-O-W bond angles of 155 and 173°, respectively.

**Table 2:** Properties of some selected Zr and W paths.

					**DW Factors** $\sigma_{p}^{2}$ **(10**^**−3**^**Å**^**2**^**)**		
**Path**	**Length (Å)**	**Amp. Ratio**	**Degeneracy**	**Legs**	**20 K**	**160 K**	**315 K**
Zr–O1–Zr	2.041	100.00	3	2	2.18	2.38	2.94
Zr–O2–Zr	2.098	92.56	3	2	2.23	2.60	3.41
Zr–W1–Zr	3.758	25.63	3	2	1.30	2.24	3.63
Zr–W2–Zr	3.871	23.67	3	2	1.18	1.65	2.53
Zr–O1–W1–Zr	3.803	44.45	6	3	1.15	1.54	2.30
Zr–O2–W2–Zr	3.874	63.22	6	3	1.18	1.62	2.48
Zr–O1–W1–O1–Zr	3.849	25.46	3	4	1.34	1.82	2.71
Zr–O2–W2–O2–Zr	3.878	42.28	3	4	1.20	1.64	2.52
W1–O4–W1	1.711	39.64	1	2	1.22	1.22	1.25
W2–O3–W2	1.733	36.04	1	2	1.27	1.27	1.32
W2–O2–W2	1.780	99.90	3	2	1.35	1.37	1.44
W1–O1–W1	1.807	100.00	3	2	1.45	1.48	1.60
W1–O3–W1	2.315	16.03	1	2	2.85	5.61	9.03
W1–Zr–W1	3.759	14.88	3	2	1.28	2.17	3.49
W1–W2–W1	4.049	4.45	1	2	1.69	4.34	7.57
W1–W2–W1	4.652	9.45	3	2	1.77	5.27	9.27
W1–O3–W2–W1	4.049	13.46	2	3	1.69	4.33	7.57
W1–O3–W2–O3–W1	4.049	8.60	1	4	1.69	4.33	7.57

*The theory used the experimental lattice constant with optimized MSRDs to avoid small phase shifts due to the 1% error in the optimized PBEsol structure*.

### 2.3. EXAFS Debye-Waller factors

The EXAFS Debye-Waller (DW) factors $W_{p}=\exp\left(-2\sigma_{p}^{2}k^{2}\right)$ provide a useful measure of damping from the correlated structural and thermal disorder in a given path. These Debye-Waller factors are essential, because they govern the exponential decay of the fine structure with increasing temperature and photoelectron energy. Our explicit calculations of $\sigma_{p}^{2}$ only include bond-length fluctuations (i.e., stretching contributions) in a given MS path, and neglect the usually negligible contributions from motion perpendicular to each bond (Poiarkova and Rehr, 1999a; Fornasini et al., 2004). Thus the MSRD for a given path is calculated in terms of averages over displacement-displacement correlation functions,

(2) $$\begin{array}{l} \sigma_{p}^{2}=\frac{1}{4}\langle \sum_{i=1}^{n_{p}}{\left[({\vec{u}}_{i}-{\vec{u}}_{i+1})\cdot {\hat{R}}_{i,i+1}\right]}^{2}\rangle , \end{array}$$

where *n*_*p*_ is the number of legs in the path and 〈⋯ 〉 denotes a thermal average. However, this MSRD neglects bond-angle fluctuations, which depend on a thermal average over the effective scattering amplitude $\langle \left|f_{\text{eff}}^{p}\left(\theta \right)\right|\rangle$ for a given path. This effect is largest for paths with forward scattering contributions, such as the Zr-O-W linkages. Expanding to 2nd order in the bond-bending angle θ and keeping only the lowest order contributions to the average, yields a damping term that can be represented as an angular Debye-Waller factor

(3) $$\begin{array}{l} W_{p}\left(\theta \right)=e^{-a\sigma_{\theta }^{2}}, \end{array}$$

$$\begin{array}{l} \sigma_{\theta }^{2}=\langle {\left(\theta -\langle \theta \rangle \right)}^{2}\rangle , \end{array}$$

where $a=-\left(1/2\right)f_{\text{eff}}^{\prime \prime }\left(\theta \right)/f_{\text{eff}}\left(\theta \right)$.

We have shown that these vibrational properties can be efficiently computed by combining DFT calculations of the dynamical matrix (DM) of lattice force constants, with a Lanczos algorithm (Vila et al., 2007). An efficient implementation based on parallelized calculations of the DMs is described in a separate paper in this collection (Vila et al., 2018), and briefly summarized below. Thermal averages such as the EXAFS mean square relative displacements $\sigma_{p}^{2}$ of a single or multiple scattering path *p* are calculated in terms of Debye integrals over the phonon projected density of states (PDOS)

(4) $$\begin{array}{l} \sigma_{p}^{2}\left(T\right)=\frac{\hbar }{2\mu_{p}}\int_{0}^{\infty }\frac{1}{\omega }\coth\left(\frac{\beta \hbar \omega }{2}\right)\rho_{p}\left(\omega \right)\;d\omega , \end{array}$$

where μ_*p*_ is the reduced mass associated with the path, β = 1/*k*_*B*_*T*, and ρ_*p*_(ω) is the PDOS for that path (Poiarkova and Rehr, 1999a,b). Higher order cumulants such as $\sigma_{p}^{\left(3\right)}$ due to anharmonic effects are neglected, which is generally a good approximation for the relatively stiff bonds considered here. In the reminder of this work the path index subscript *p* is ignored unless needed for clarity.

The key system-dependent ingredient for $\sigma_{p}^{2}$ in Equation (4) is the PDOS ρ_*p*_(ω). Following Poiarkova and Rehr (1999a,b) the unit-normalized PDOS is obtained from the imaginary part of path specific matrix elements of the lattice dynamical Green's function

(5) $$\begin{array}{l} \rho_{p}\left(\omega \right)=-\frac{2\omega }{\pi }\text{Im}\langle p\left|\frac{1}{\omega^{2}-\text{D}+i\epsilon }\right|p\rangle . \end{array}$$

Here |*p*〉 is a Lanczos seed vector representing an initial normalized, mass-weighted displacement along path *p*, and **D** is the dynamical matrix

(6) $$\begin{array}{l} D_{jl\alpha ,j\prime l\prime \beta }={\left(M_{j}M_{j^{\prime }}\right)}^{-1/2}\;\frac{\partial^{2}U[\{u_{jl\alpha }\}]}{\partial u_{jl\alpha }\partial u_{j\prime l\prime \beta }}, \end{array}$$

where *U* is the total internal energy of the system as a function of lattice coordinates, *u*_*jlα*_ is a displacement of atom *j* in unit cell *l* along the direction α, and *M*_*j*_ is the mass of atom *j*. The phonon Green's function is obtained from a continued fraction representation with parameters derived from the Lanczos algorithm (Haydock, 1980; Deuflhard and Hohmann, 1995). This yields a many-pole representation of the PDOS

(7) $$\begin{array}{l} \rho_{p}\left(\omega \right)=\overset{N_{\nu }}{\underset{\nu =1}{\sum }}w_{\nu }\;\delta \left(\omega -\omega_{\nu }\right). \end{array}$$

where *w*_ν_ and ω_ν_ are the weights and frequencies of the *N*_ν_ poles, respectively. Typically only a few tens of poles are needed to converge calculations of $\sigma_{p}^{2}$.

## 3. Calculations

### 3.1. Debye-Waller factors

The computational details of the Debye-Waller factor calculations are similar to those in Vila et al. (2018). Briefly, structural optimizations and DM calculations were performed with VASP (Kresse and Hafner, 1993, 1994; Kresse and Furthmüller, 1996a,b; Kresse and Joubert, 1999) using PAW pseudopotentials (Kresse and Joubert, 1999) with the PBEsol exchange-correlation functional (Perdew et al., 2008), and an energy cutoff of 350 eV; a *k*-point grid of 8 × 8 × 8 was used to achieve converged results. The lattice constant of the cell and internal reduced coordinates of the atoms in it were optimized while preserving initial lattice symmetry for computational efficiency. This DFT optimization yields a unit cell expanded by about 1% from the experimental value (see Table 1) with internal reduced coordinates that are in good agreement with experiment. Given that the internal reduced coordinates do not change with volume until the phase transition at about 430 K (Evans et al., 1999), and that the change of force constants with temperature is small in the range studied, we use the 0 K optimized structure to compute the DM. The DM is obtained using a parallelized finite difference approach with a three point centered stencil with 0.015 Å atomic displacements. The structure of the unit cell as well as the dynamical matrix are available as Supplementary Material.

**Figure 2:**
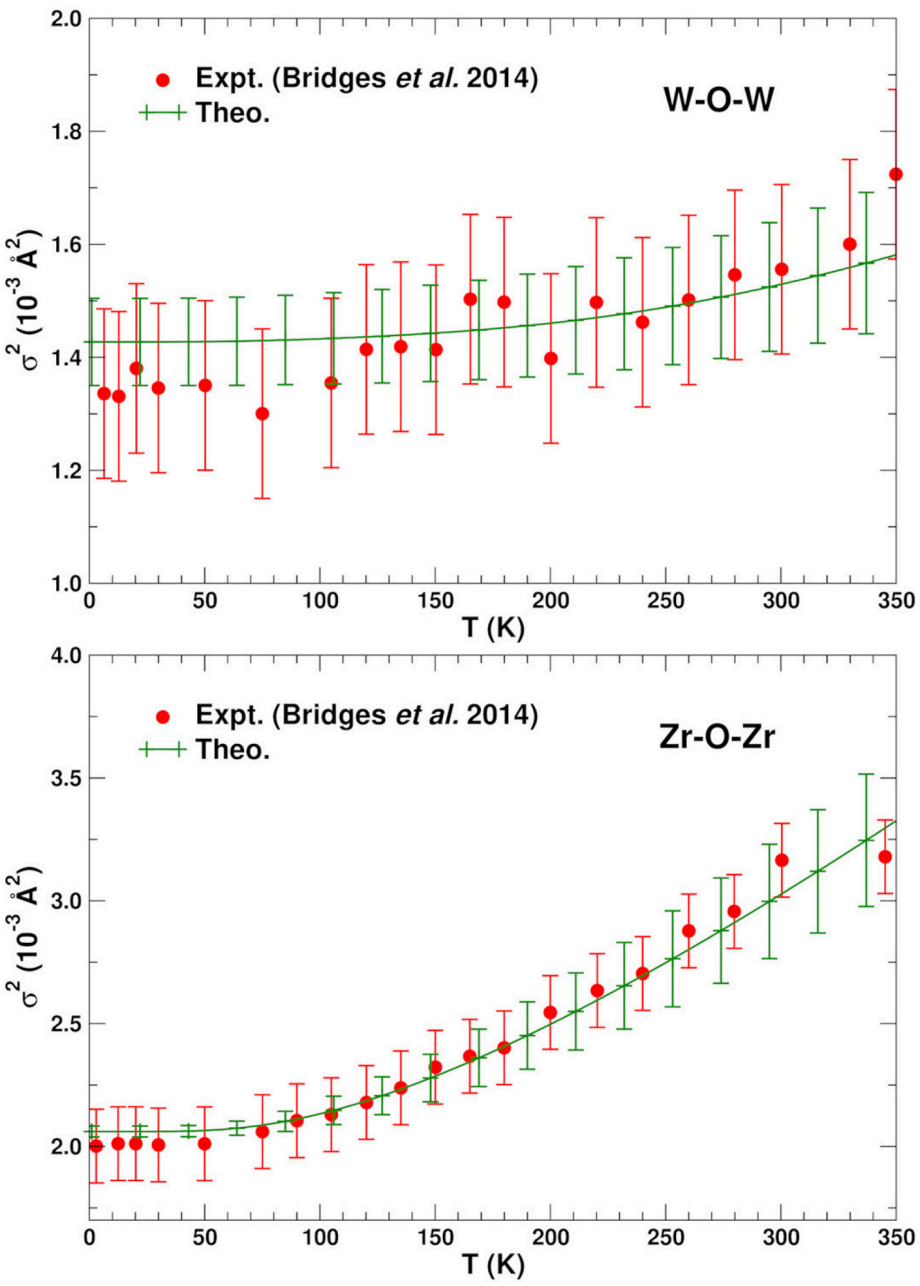
Comparison of the experimental (Bridges et al., 2014) and average theoretical single scattering MSRD $\sigma_{p}^{2}$ for the W–O–W and Zr–O–Zr in ZrW_2_O_8_. The theoretical values have been shifted vertically to account for experimental static disorder.

**Figure 3:**
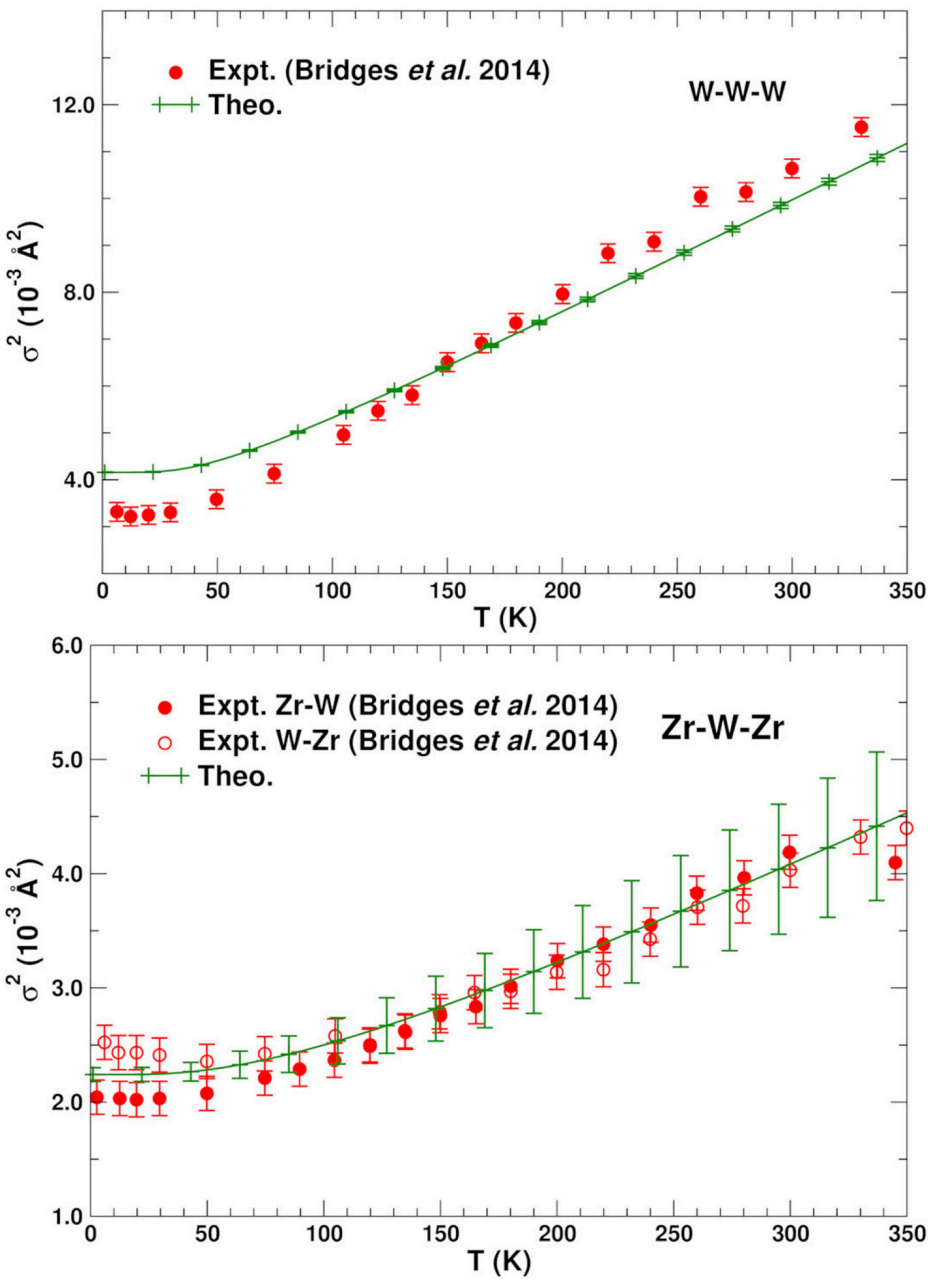
Comparison of the experimental (Bridges et al., 2014) and theoretical mean single scattering MSRD $\sigma_{p}^{2}$ for the W–W–W and Zr–W–Zr bonds in ZrW_2_O_8_. The theoretical values have been shifted vertically to account for experimental static disorder.

Figures 2, 3 show the average calculated MSRDs for the various SS paths (W–O–W, Zr–O–Zr, W–W–W, and Zr–W–Zr), together with comparisons to experiment. Since only bond-stretching contributions are included $\sigma_{p}^{2}$ these results have been shifted to account for the measured structural disorder $\sigma_{static}^{2}$ which contributes additively, i.e., $\sigma^{2}=\sigma_{\text{static}}^{2}+\sigma_{\text{stretch}}^{2}$. The required shift was estimated by computing the mean absolute deviation between the theoretical and experimental MSRDs over the available temperature range. The static disorder has both physical and methodological origins. The physical static disorder arises mostly from the presence of defects and distortions which are intrinsic in materials like ZrW_2_O_8_, while the EXAFS analysis method introduces apparent disorder by collecting paths of closely similar lengths into a single contribution with a Debye-Waller factor that reflects the length variations. Thus, for the W–O–W and Zr–O–Zr paths, which correspond to strong, well-defined bonds, the static component is very small. For the W–W–W and Zr–W–Zr paths, on the other hand, the static disorder is significant since these paths connect different WO_4_ and ZrO_6_ units through a variety of soft bonds. Note that the bond stretching contributions to $\sigma_{p}^{2}$ for these paths only involve planar fluctuations of the atoms ${\vec{u}}_{i}$ in the path, and that contributions to $\sigma_{stretch}^{2}$ from transverse displacements are generally negligible, even for substantial transverse O-displacements. Nevertheless, angular damping *W*_*p*_(θ) may be important for paths like Zr-O-W involving forward focusing (see below). Clearly the shifted MSRDs agree well with experiment (Bridges et al., 2014). Their associated Einstein temperatures Θ_*E*_ = ℏω_*E*_/*k*_*B*_, where ω_*E*_ is the mean vibrational frequency of the PDOS for a given path, are given in Table 3. Again the agreement with experiment is satisfactory, with the largest deviations occurring for the W–W–W paths. These paths are the longest ones and thus fall in the upper range of paths lengths that can be adequately described with the present simulation cell. The slight underestimation of the MSRD for these paths would likely be reduced with a larger simulation cell which would improve the representation of the low frequency- long spatial wavelength region of the phonon density of states. The computed MSRDs are consistent with physically expected interaction strengths. For example, the W–O and Zr–O bonds are the strongest, followed by the weak, long-range interactions between the Zr–W and W1–W2 pairs.

**Table 3:** Comparison of the theoretical and experimental (Bridges et al., 2014).

	Θ_*****E*****_		κ_*****E*****_	
**Path**	**Theory**	**Expt**.	**Theory**	**Expt**.
W–O–W	1,033	878 ± 68	447	323 ± 2
W–W–W	146	129 ± 4	56	44 ± 1
Zr–O–Zr	588	608 ± 36	134	143 ± 1
W–Zr–W	281	299 ± 12	137	155 ± 1
Zr–W–Zr	281	274 ± 16	137	130 ± 1

*Einstein temperatures Θ_E_ = ℏω_E_/k_B_ (in K) and effective force constants $\kappa_{E}=\mu {\omega_{E}}^{2}$ associated with the dominant single scattering paths*.

**Figure 4:**
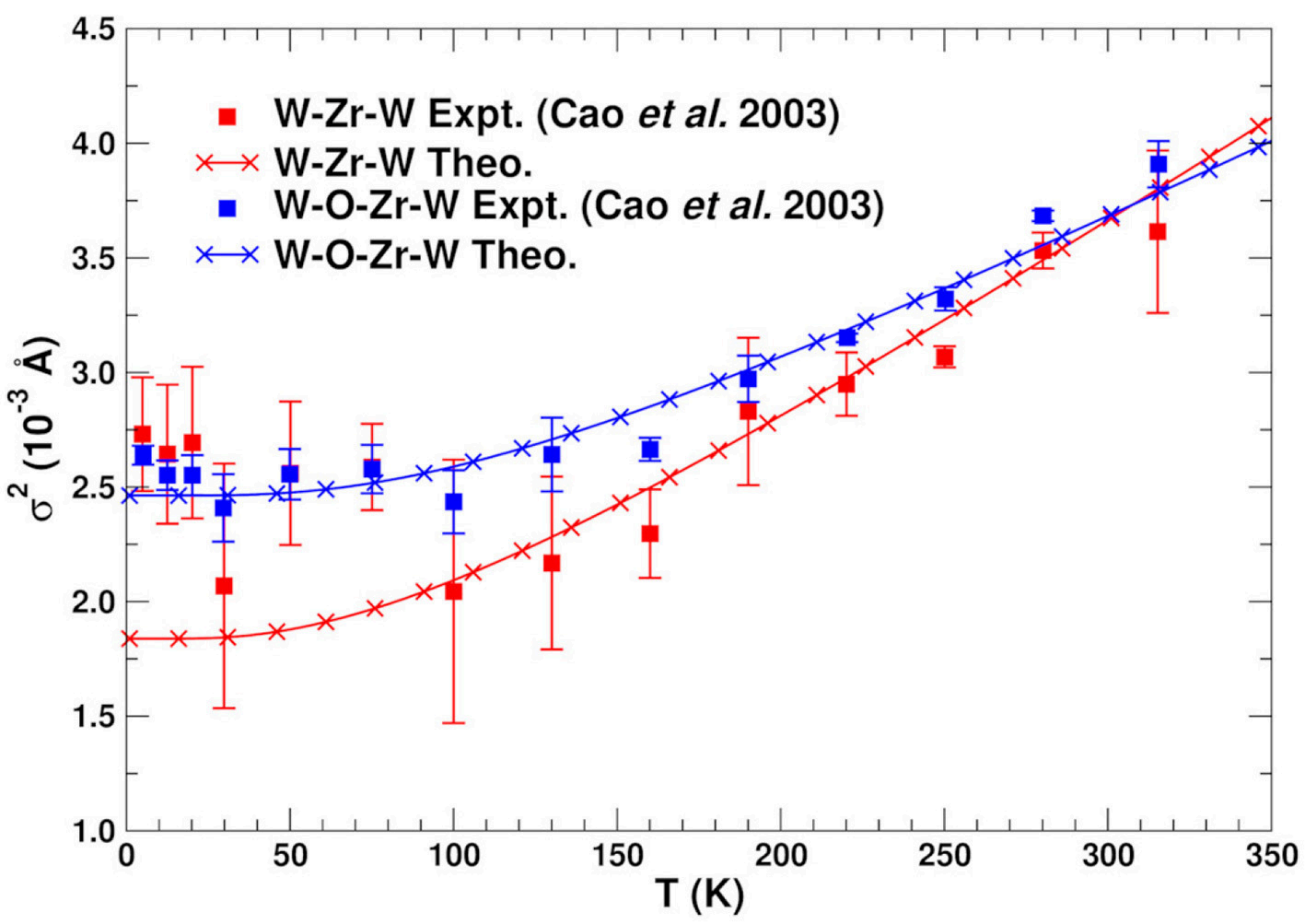
Comparison of the experimental (Cao et al., 2003) and theoretical MSRD σ^2^ for the SS and MS paths associated with the Zr–W linkage in ZrW_2_O_8_. The theoretical values are shifted vertically to account for experimental static disorder.

**Figure 5:**
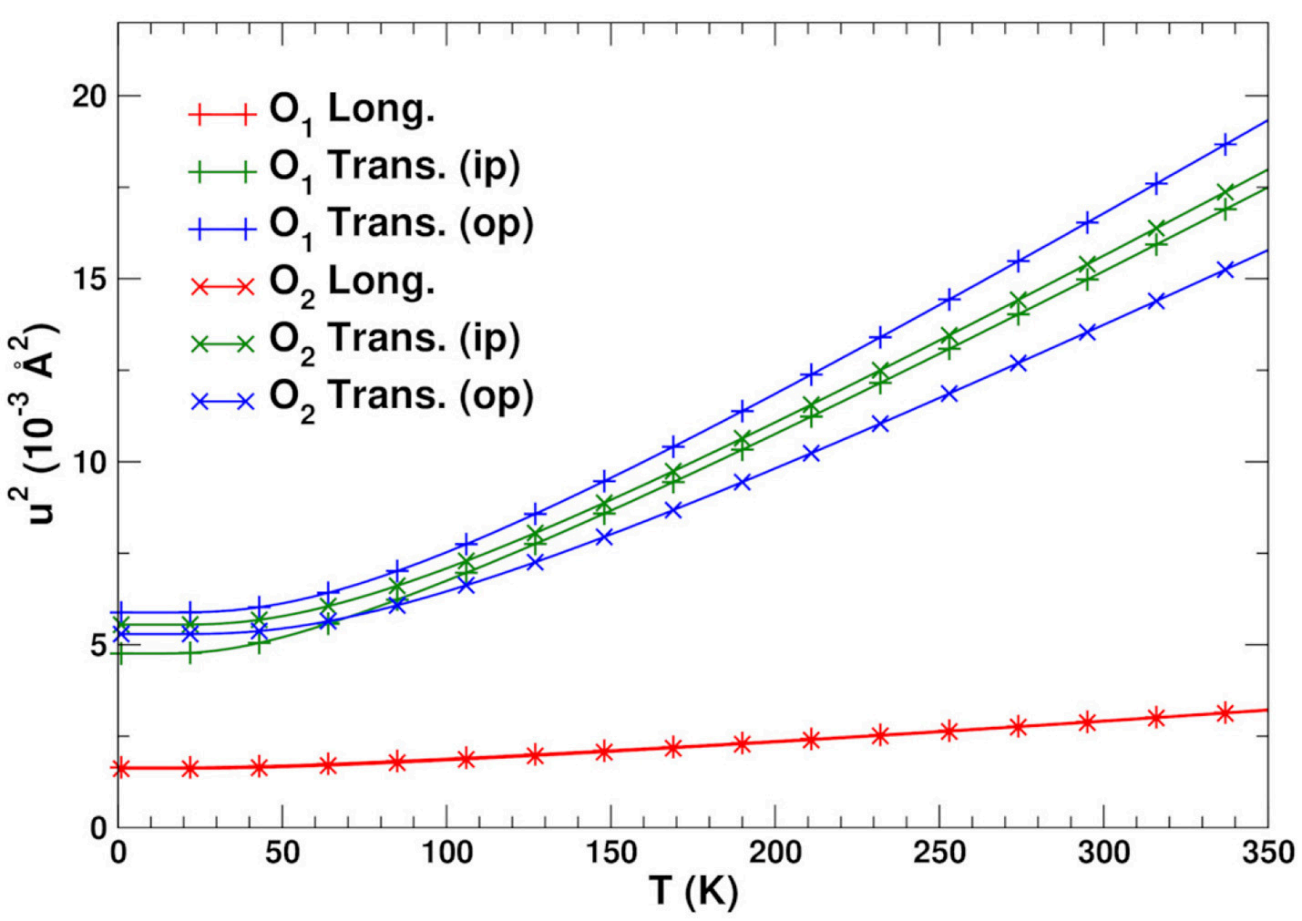
Longitudinal and transverse MSDs *u*^2^ for the O1 and O2 bridging atoms.

As noted above the most relevant paths for EXAFS studies of NTE are those involving the Zr and W atoms. Table 2 summarizes the structural properties of these paths, as well as their MSRDs (without static disorder corrections) at three temperatures. Note that the MSRDs for the SS and MS 173° paths are nearly identical, since they are nearly collinear and the push-pull distortions of the Zr–O and W–O bonds essentially cancel. For the 155° linkage paths, the MS MSRDs are also similar again because they involve near-neighbor bond distortions. The only exception is the Zr–W1–Zr path that is slightly softer. This path involves the nearly-independent coupling between the Zr and W atoms, which is dominated by the distortion of 2 Zr–O bonds and all 4 of the W–O bonds. This suggests that the MSRD of Zr–W1–Zr paths is larger because the W1–O1 bonds are longer, and thus weaker, than the W2–O2 ones. Figure 4 shows a comparison of the average theoretical DW factors for the Zr–W–Zr and Zr–O–W–Zr paths with experiment (Cao et al., 2003). Clearly our results match closely except at low temperature, where the experimental data also has significant errors. It is interesting to note that the larger slope for the Zr–W–Zr path arises from the Zr–W1–Zr contribution. Overall, the results in Figure 4 and Table 2 show that the length-fluctuations $\sigma_{p}^{2}$ of these paths are only weakly temperature dependent, and thus are “stiff,” consistent with the interpretation of Bridges et al. (2014). The stiffness of a given SS or MS path linkage is characterized by the effective spring constant κ_*p*_ for the DW factor of a given path *p*. It is reflected by its temperature dependence which varies as *k*_*B*_*T*/κ_*p*_ at high *T*. More precisely, $\kappa_{p}=\mu_{p}{{\bar{\omega }}_{p}}^{2}$ where $1/{\bar{\omega }}_{p}^{2}=\langle 1/\omega^{2}\rangle$ is the inverse 2nd moment of the PDOS. Thus the Zr–O–W linkage is considered stiff because for a large κ_*p*_, the associated path DW factor varies weakly with temperature with a small slope 1/κ_*p*_. Note however, that this weak temperature dependence does not preclude the existence of large transverse fluctuations of the O atoms in the linkages. Our calculations of the DM show that the transverse fluctuations perpendicular to the Zr–O–W plane have much smaller spring constants (Vila et al., 2018) and, hence, substantial larger values of *u*^2^ for the O atom in the linkage (see Figure 5).

### 3.2. Atomic displacements

Figure 5 shows the longitudinal and transverse mean square displacements (MSD) *u*^2^ for the O1 and O2 linkage atoms. The in-plane transverse direction here is defined as the bisector of the Zr–O–W angle. The longitudinal and transverse out-of-plane directions are defined as the in- and out-of-plane perpendicular directions to this bisector, respectively. Although the 155° linkage is more asymmetric and therefore might be expected to be anisotropic, both types of linkages have similar transverse MSDs. In contrast the longitudinal motion has a relatively small, high-frequency amplitude with a large associated Einstein temperature of 600 K, as expected from the distortion along the stiff W–O and Zr–O bonds. Due to the weaker force constants associated with the bond bending (Vila et al., 2018), however, the transverse motion has an Einstein temperature of about 250 K. At T = 300 K this results in *rms* amplitudes *u* ≈ 0.12 Å. This corresponds roughly to linkage angle distortions of about δθ ≈ 6°, which are more than twice the longitudinal fluctuations at the same temperature. Although these transverse distortions are substantial, they have little effect on the bond stretching MSRDs (Poiarkova and Rehr, 1999a; Fornasini et al., 2004). For the SS paths, this is easy to see, since for a bond of length *R*, the perpendicular contribution from each atom is $\sigma_{⊥}^{2}\sim {\left(u^{2}/2R\right)}^{2}$. For the linkage bonds with *R*~ 2 Å and *u*~ 0.12 Å, we estimate that $\sigma_{⊥}^{2}\sim$ 0.02 × 10^−3^ Å^2^, which is an order of magnitude smaller than the typical error margins for the experimental MSRDs. A similar approach can be used for the multiple scattering paths, where the contributions of all atoms in the path need to be added. On the other hand the angular DW factors *W*_*p*_(θ) may still contribute to the damping of the EXAFS from these paths.

**Figure 6:**
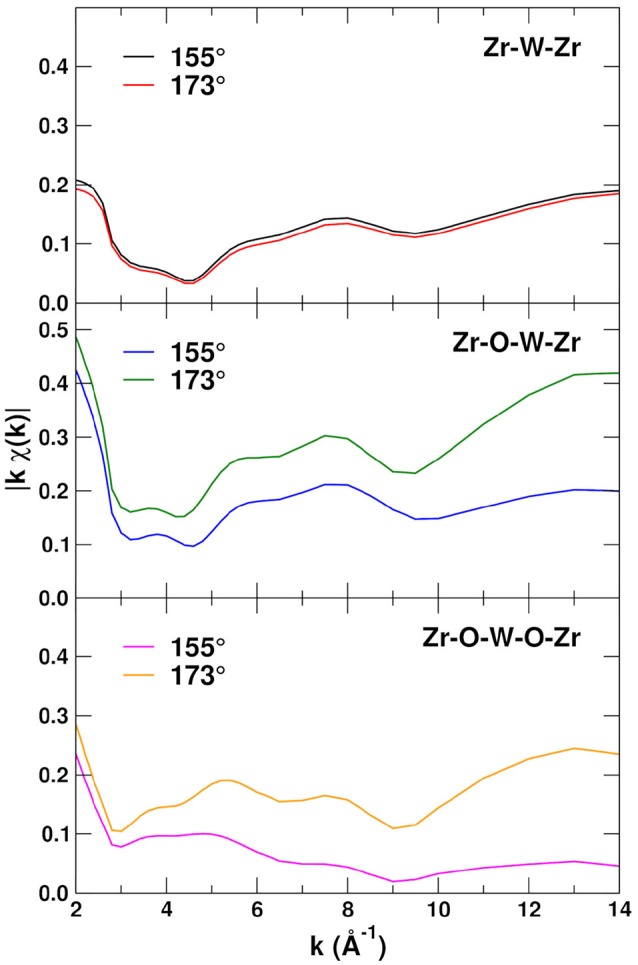
EXAFS path amplitudes $\left|k{\tilde{\chi }}_{p}\left(k\right)\right|$ for the Zr-W-Zr single-scattering and Zr-W-O-Zr and Zr-O-W-O-Zr MS paths with linkage angles 155 and 173°.

In the amplitude-phase representation of EXAFS the magnitude of each path contribution is given by $\left|{\tilde{\chi }}_{p}\left(k\right)|=|f_{\text{eff}}^{p}/kR^{2}\right|$, which eliminates the oscillations and thus emphasizes the amplitudes of the various paths. Figure 6 shows the amplitudes $\left|k{\tilde{\chi }}_{p}\left(k\right)\right|$ for the 155 and 173° Zr–O–W linkages, including the Zr–W–Zr SS and Zr–W–O–Zr and Zr–O–W–O–Zr MS paths. Note that the amplitudes $\left|{\tilde{\chi }}_{p}\left(k\right)\right|$ do not include the Debye-Waller factors and thus have no temperature dependence. The Zr–W–Zr paths are not affected by the angle of the linkage for the Zr–W–O–Zr and Zr–O–W–O–Zr paths, also the amplitudes for the 173° linkage paths, are on average about twice as large as for the 155° paths. These differences are due to the angular dependence and forward scattering enhancement in $f_{\text{eff}}^{p}$ and are particularly strong for bond angles close to 180° and at high *k*.

### 3.3. EXAFS spectra

**Figure 7:**
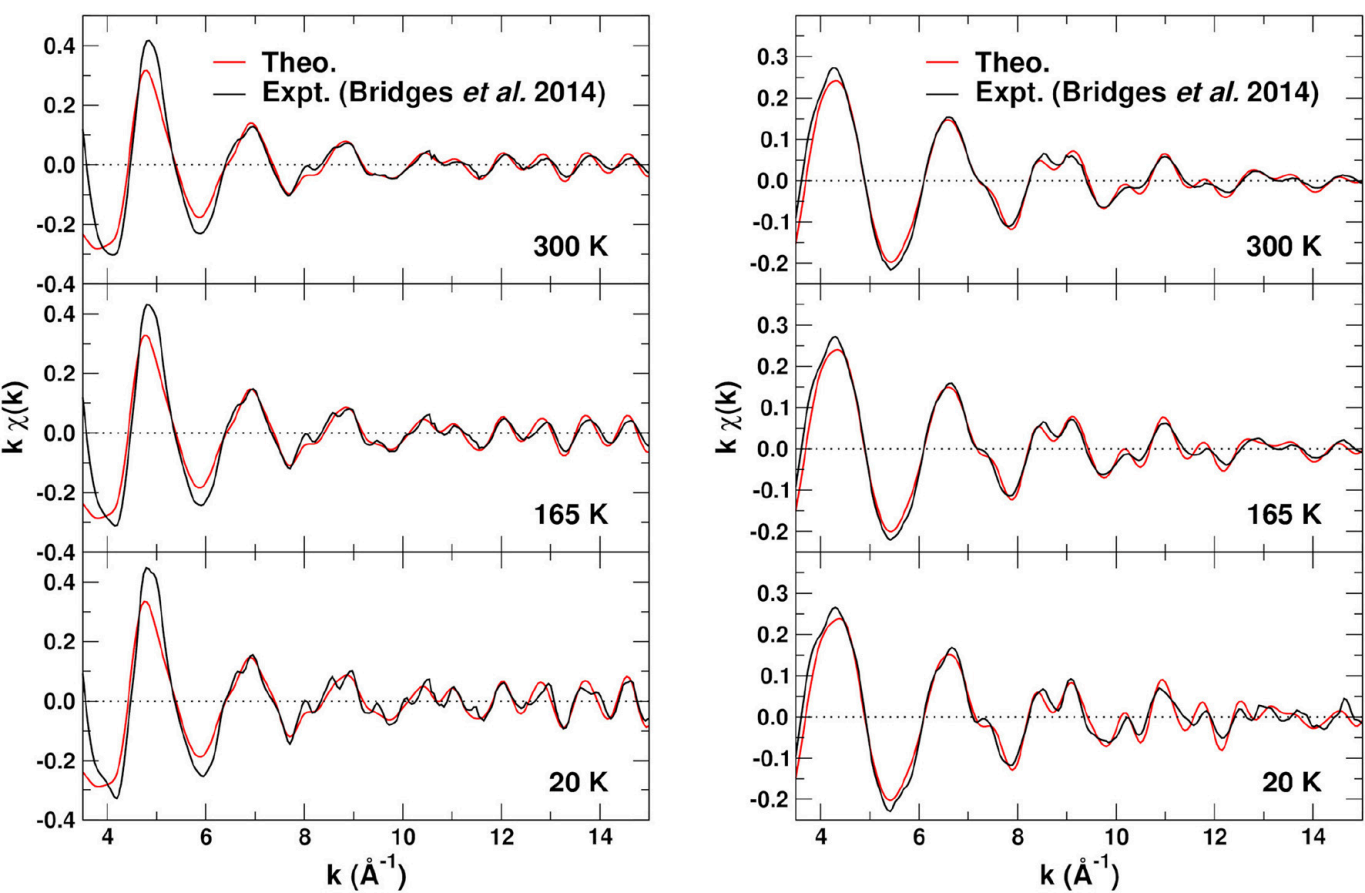
Comparison of the experimental (Bridges et al., 2014) and theoretical EXAFS fine structure *kχ*(*k*) at 20, 165, and 300 K for the Zr K edge (left) and the W L_3_ edge (right). The theory used the experimental lattice constant with optimized MSRDs to avoid small phase shifts due to the 1% error in the optimized PBEsol structure.

To assess the overall quality of our EXAFS calculations, Figure 7 shows comparisons of the experimental (Bridges et al., 2014) fine structure *kχ*(*k*) at 20, 165, and 300 K for the Zr K- and W-L_3_ edges to those obtained with FEFF. The theoretical results used optimized MSRDs with bond distances reduced by 1% to correct the overestimate of the lattice constant by DFT/PBEsol calculations. Our EXAFS calculations were obtained by including all SS and MS paths of up to 5.5 Å and *n*_*p*_ = 4 scattering legs, and by removing all path screening filters from FEFF. The experimental results (Bridges et al., 2014) find that the many-body amplitude factor $S_{0}^{2}$ in Equation (1) is very close to 1, thus we use $S_{0}^{2}=1$ in the EXAFS simulations. Clearly the agreement is quite satisfactory for the EXAFS range *k* ≳ 5 Å^−1^, below which the path expansion approach is less appropriate and atomic background substraction artifacts are possible. The small, high frequency contribution noticeable in the Zr K-edge signal at 20 K corresponds to ~ 6.5 Å Zr–Zr–Zr paths which are not included in our simulation cell.

**Figure 8:**
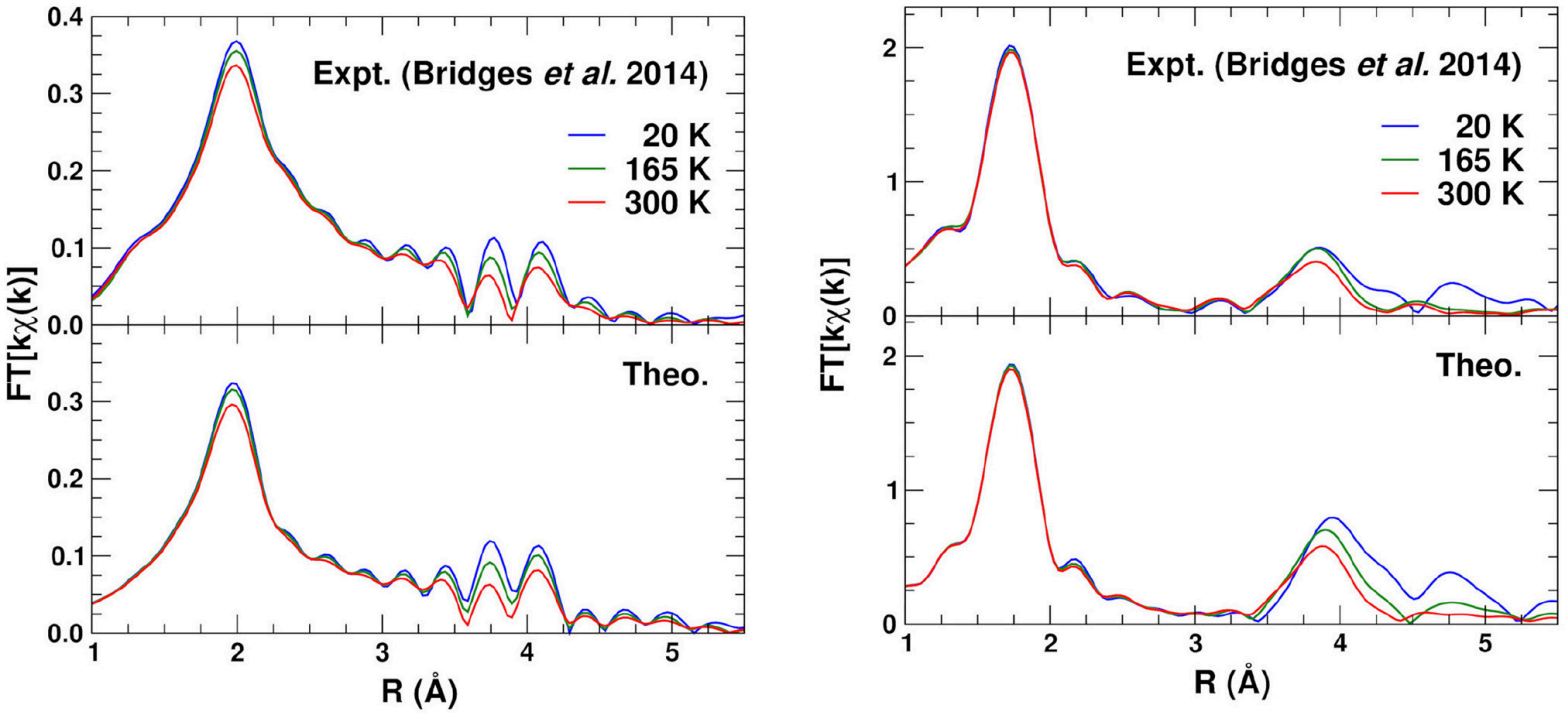
Comparison of the Fourier transforms of the experimental (Bridges et al., 2014) and theoretical *kχ*(*k*) (Figure 7) at 20, 165, and 300 K for the Zr K edge (left) and the W L_3_ edge (right).

Finally Figure 8 shows the *phase-corrected* Fourier transforms (FTs) of the *kχ*(*k*). For these transforms the central atom theoretical phase-shift 2δ_*l*_ common to all paths is subtracted before being transformed (Rehr et al., 2009), resulting in peak positions closer to their geometrical locations. Again, the overall agreement is good, except for differences in the heights of some of the peaks, due probably to static and angular disorder in the DW factors. For the Zr absorber, the FTs clearly show the Zr–O peak at about 2 Å, and the different linkage paths between 3.5 and 4.2 Å.

A potentially important issue is the effect of the Zr–O–W bond angle fluctuations during the complex low energy vibrations of the system, particularly for the Zr–O2–W2 linkage. The amplitudes of the MS peaks for the Zr–O–W linkages vary significantly with bond angle due to forward scattering through the O atom. While *f*_eff_(θ) is strongly peaked in the forward direction and varies substantially with θ, the angular Debye-Waller factor in Equation (3) depends on the fluctuations $\sigma_{\theta }^{2}\approx 0.01$ rad^2^. To estimate these effects we computed the average χ(*k*) for the Zr–O1–W1 and Zr–O2–W2 linkages over the distribution of angular distortions induced by mean *u* amplitues of about 0.12 Å. We find that with distortions of this amplitude the absolute change in χ(*k*) is only about 3 and −6% for the Zr–O1–W1 and Zr–O2–W2 linkages, respectively. This explains the good agreement between the calculations without including such averaging, and the data for the Zr edge in Figure 8. The actual situation can be more complicated, however, since the polyhedra can rotate as well as translate and a full treatment that takes this correlated motion into account would be desirable.

## 4. Summary and conclusions

We have shown that theoretical calculations of structure, vibrational properties and the EXAFS of ZrW2O8 agree well with experiment (Bridges et al., 2014). The structural parameters are calculated using the DFT plane-wave pseudopotential VASP code with DFT/PBEsol while vibrational properties are obtained using a Lanczos algorithm and the phonon Green's function approach. The EXAFS calculations depend sensitively on these structural and vibrational properties. The fact that the calculations of the MSRDs for the most important SS and MS paths agree well show that an accurate treatment of correlated atomic motion is essential to understand the dynamic disorder. The small differences can likely be accounted for by static disorder and angular DW factors. For example, forward focusing effects in the Zr-O-W linkages are particularly important in these calculations. For example, the nearly collinear 173° paths contribute more to the amplitudes, due to enhanced forward scattering. Our calculations also show that transverse O fluctuations are substantial, consistent with results of our companion study (Vila et al., 2018), which imply that NTE is driven in part from the negative Grüneisen parameters of the low-frequency O modes. Thus while the effect of transverse fluctuations on the MSRDs is negligible, they may contribute to the angular DW factors. Our calculations also ignore anharmonic effects; although such effects are generally associated with thermal expansion (Frenkel and Rehr, 1993), this is a reasonable approximation for the dominant SS and MS paths with relatively stiff bonds. Overall our theoretical calculations yield results consistent with experiment for ZrW_2_O_8_ for a broad range of properties. In particular they show that both transverse motion and correlated atomic displacements are crucially important to understand the dynamic structure of this material.

## Author contributions

FV co-developed the theory, was the main developer of the specialized software used in the analysis, and performed most of the simulations. JS contributed to the initial simulations. JK performed part of the analysis (Fourier Transforms). JR co-developed the theory and contributed to the analysis. FB provided EXAFS data and contributed to the analysis.

### Conflict of interest statement

The authors declare that the research was conducted in the absence of any commercial or financial relationships that could be construed as a potential conflict of interest.
